# Clinical Values of the Identified Hub Genes in Systemic Lupus Erythematosus

**DOI:** 10.3389/fimmu.2022.844025

**Published:** 2022-06-09

**Authors:** Lu Xiao, Feng Zhan, Shudian Lin

**Affiliations:** Department of Rheumatology, Hainan General Hospital (Hainan Affiliated Hospital of Hainan Medical University), Hainan, China

**Keywords:** systemic lupus erythematosus, bioinformatics analysis, biomarker, receiver operating characteristic curve, SLEDAI

## Abstract

**Objective:**

This study was conducted to identify the biomarkers and mechanisms associated with systemic lupus erythematosus(SLE) at a transcriptome level.

**Methods:**

Microarray datasets were downloaded, and differentially expressed genes (DEGs) were identified. Enrichment and protein–protein interaction networks were analyzed, and hub genes were discovered. The levels of top 10 hub genes were validated by another dataset. The diagnostic accuracy of the hub genes was evaluated with the area under the curve of the receiver operating characteristic curve (ROC-AUC). The odds ratios (OR) and 95% confidence intervals (CI) of the relationship between clinical manifestations and hub genes were estimated with multivariable logistic regression. The relationships between the expression levels of the 10 identified hub genes and SLEDAI scores were subjected to linear correlation analysis. Changes in the expression levels of the hub genes during patient follow-up were examined through one-way repeated measures ANOVA.

**Results:**

A total of 136 DEGs were identified. Enrichment analysis indicated that DEGs were primarily enriched in type I interferon-associated pathways. The identified hub genes were verified by the GSE65391 dataset. The 10 hub genes had good diagnostic performances. Seven (except IFI6, OAS1 and IFIT3) of the 10 hub genes were positively associated with SLEDAI. The combination models of IFIT3, ISG15, MX2, and IFIH1 were effective in diagnosing mucosal ulcers among patients with SLE. The expression levels of IRF7, IFI35, IFIT3, and ISG15 decreased compared with the baseline expression (not significantly).

**Conclusions:**

In this work, the clinical values of the identified hub genes in SLE were demonstrated.

## Introduction

Systemic lupus erythematosus (SLE) is one of the autoimmune diseases predominantly affecting young women and involving multiple organs (1). It is characterized by a broad spectrum of clinical symptoms from mild fatigue and rash to severe, life-threatening organ damage, such as hematologic abnormalities, renal injury, and neuropsychiatric involvement (2). The pathogenesis of SLE focuses on abnormal immune system activating and attacking healthy cells and tissues throughout the body (3). SLE is prevalent among Asians, Hispanics, and Americans. The incidence of SLE in China is approximately 30 per 100,000 (4).Due to the huge impact on patients’ quality of life, uncovering the underlying molecular characteristics and mechanism of SLE is very important to discover reliable biomarkers for diagnosis and effective therapy.

At present, transcriptomic and microarray analyses have been widely used to explore promising biomarkers to improve the diagnosis and treatment of disease at the genome level (5–7). Numerous bioinformatic studies have demonstrated different abnormal expression levels of genes associated with the development of SLE. Lu et al. reported that the methylation level of IFI44L promoter could distinguish patients with SLE from healthy people (8). IFIT1 expression was also proven to be associated with podocytes damage and be capable of suppressing some proteins essential to glomerular filtration in MRL/lpr mice (9). Abnormal elevation in IFIT3 was demonstrated to be associated with overactive cyclic GMP-AMP synthase/stimulator of interferon (IFN) genes signaling in SLE monocytes (10). Moreover, other bioinformatic studies have reported abnormal high levels of IFI27, RPL26L1, FBXW11, FOXO1, and SMAD7 in patients with SLE (7, 11). Therefore, exploring the accurate molecular targets included in the occurrence and progression of SLE is imperative. Through the combination of microarray and bioinformatics analyses, exploring potential key genes and pathway networks that are closely related to the development of SLE is possible.

In this study, microarray datasets GSE49454 and GSE20864 for patients with SLE and healthy controls (HCs) were first downloaded from the Gene Expression Omnibus (GEO) database. A total of 178 patients with SLE and 65 HCs were included. The data were integrated and reanalyzed. A total of 136 common differentially expressed genes (DEGs) were identified, including 122 upregulated genes and 14 downregulated genes. DEGs were clustered with Gene Ontology (GO) and Kyoto Encyclopedia of Genes and Genomes (KEGG) pathway enrichment analysis. Furthermore, a protein–protein interaction (PPI) network was constructed using the online tool STRING, and Cytoscape was used to identify cluster modules and hub genes related to SLE. Subsequently, the selected hub genes were validated using the GEO dataset GSE65391. The diagnostic accuracy of the identified hub genes for SLE was evaluated with the area under the curve of the receiver operating characteristic curve (ROC-AUC). The odds ratios (OR) and 95% confidence intervals (CI) of the relationship between clinical manifestations and hub genes were estimated with multivariable logistic regression. The diagnostic accuracy of the identified hub genes in diagnosing different clinical manifestations was also demonstrated by ROC-AUC. The relationships between the hub gene expression and the SLE disease activity index (SLEDAI) were analyzed through linear correlation. Repeated measures ANOVA was applied to compare the expression levels of the hub genes in patients with SLE at three follow-ups.

## Materials and Methods

### Data Collection

‘‘Systemic lupus erythematosus’’ was used as the key word to search expression profiling of SLE in GEO database, which is a public repository database (12). Studies that met the following criteria were included: 1) whole genome expression data of SLE, 2) datasets containing more than five samples, and (3) datasets containing complete information about the samples. Finally, two datasets GSE49454 (GPL10558) and GSE20864 (GPL1291), including 178 SLE samples and 65 HC samples, were selected as test sets (13, 14). One dataset GSE65391 (GPL10558), which included 137 SLE samples and 53 HC samples, was selected as the validation set. Basic information, including platform, samples, source tissue, age, sex, attribution, and diagnostic criteria, are listed in **Supplementary Table 1** (15). A flowchart for the overall study is shown in **Supplementary Figure 1**.

### Identification of DEGs

The raw expression data of GSE49454 and GSE20864 were analyzed. The DEGs between patients with SLE and HCs were obtained through the online tool GEO2R, an R-based web application that helps users analyze GEO data. The GEO2R back end uses established Bioconductor R packages to transform and analyze GEO data and presents results as a table of genes ordered by significance (16). After selecting “Analyse with GEO2R” on series record GSE49454 and GSE20864, a table of the samples in the two studies and their descriptions were presented. In this case, two sample groups including “SLE” and “HC” are defined, and different samples are assigned to each group. Then, we click the “Download full table “button to retrieve a table of the DEGs with statistics and gene annotation (17). Adjusted P value < 0.01 was set as the threshold to screen the DEGs. The online tool Draw Venn Diagram (http://bioinformatics.psb.ugent.be/webtools/Venn/) was applied to detect the overlapping DEGs between the two datasets.

### Functional and Pathway Enrichment Analysis

GO enrichment and KEGG pathway analysis for the identified DEGs were performed by R packages (clusterProfile, ggplot2 and GOplot) (18). The ClusterProfile package was applied to analyze the DEGs. The Ggplot2 and GOplot packages were used for visualization of the results.

### Construction of PPI Network

The common DEGs were analyzed by using the online tool STRING (https://string-db.org) to construct the PPI network (19).In STRING database, the interaction score is used to set the cut-off standard. The confidence cut-off for showing interaction links can be set to highest (0.9), high (0.7), medium (0.4), and low (0.15). In general, the interaction scores in STRING are meant to express an approximate confidence, on a scale of zero to one, of the association being true, given all the available evidence. Usually, medium confidence is the default setting interaction score as a combined score >0.4 (19, 20). Therefore, the cut-off standard was set as a combined score >0.4 in our study. Then, the results were visualized by Cytoscape software. Molecular Complex Detection (MCODE) V1.5.1, which is a plug-in of Cytoscape, was applied to identify significant modules (MCODE score ≥4) (21). Moreover, the hub genes were chosen by Cytohubba, which is another plug-in of Cytoscape, based on a high number of associations with other genes in the PPI network (22). CytoHubba provides 11 topological analysis methods including Degree, Edge Percolated Component, Maximum Neighborhood Component, Density of Maximum Neighborhood Component, Maximal Clique Centrality (MCC), and six centralities (Bottleneck, EcCentricity, Closeness, Radiality, Betweenness, and Stress) based on shortest paths. Among the eleven methods, MCC, has a better performance on the precision of predicting essential proteins from the PPI network according to previous study (22). In order to increase the sensitivity and specificity, MCC was used to discover hub genes.

### Follow-Up Information of Patients in GSE65391

GSE65391 is a dataset that includes the clinical and laboratory indexes of 137 patients and 53 HCs. It also records follow-up information, including different laboratory indicators and gene expression levels, in detail. Each patient has an ID number in the dataset, such as “whole blood-SLE-99-V1-SLE-1,” where V1 stands for the first visit and the follow-up information is obtained by searching the ID of each patient. The expression level of each hub gene was recorded in the EXCEL file.

### Statistical Analysis

Statistical analysis was performed with Rstudio software and IBM SPSS Statistics 22 (SPSS, Inc., Chicago, IL, USA). Continuous variables were presented as the mean *±* standard deviation (SD). The expression levels of 10 identified hub genes were validated by GSE65391 using Mann–Whitney u test, as the samples do not satisfy the normality test. The ROC-AUC was used to compare the diagnostic performance of different hub genes. The associations between clinical manifestations and the expression levels of different hub genes were analyzed by multivariable logistic regression. Linear correlation analysis was performed by the software GraphPad Prism 7 to determine the relationship between SLEDAI and the expression levels of the 10 hub genes. One-way repeated measures ANOVA was conducted to analyze the variation in the expression levels of hub genes during follow-up, with the expression levels of hub genes as the independent variable and one visit as the dependent variable. For a significant factor effect, a *post-hoc* test was performed with Bonferroni adjustment (IBM SPSS Statistics 22). GraphPad Prism 7 was also used to show the hub gene expression of patients with SLE on different visits. P < 0.005 was considered statistically significant after correction of multiple hypothesis testing for 10 genes.

## Results

### Identification of Common DEGs

**Figure 1:**
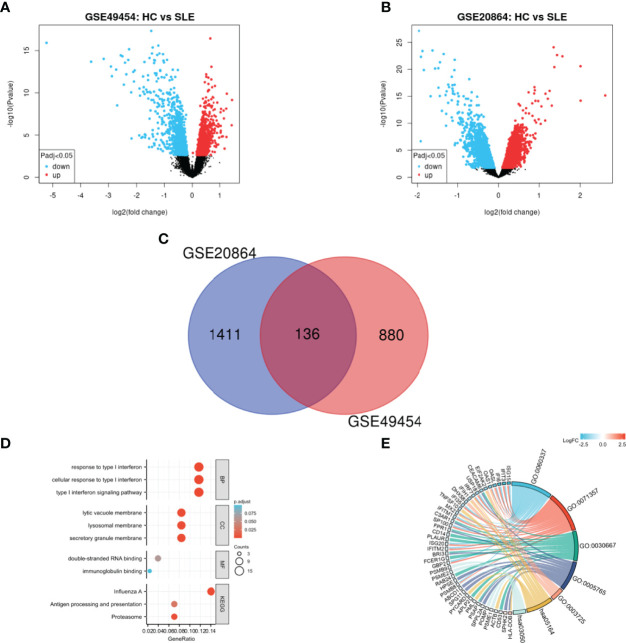
Identification and functional enrichment of DEGs. **(A)** Volcano plot of GSE49454, **(B)** Volcano plot of GSE20864, and **(C)** Venn diagram of common DEGs from the two datasets. Red and blue data points represent the upregulated and downregulated genes, respectively. **(D)** Histogram and **(E)** chordal graph of GO enrichment and KEGG pathway analysis of the overlapping DEG.

By analyzing the differences between SLE and HCs with two-group comparison, 1547 and 1016 DEGs from GSE20864 and GSE49454 were identified, respectively. The volcano plots of the two gene datasets are shown in **Figures 1A, B**. After these DEGs were integrated by employing bioinformatic analysis, a total of 136 common DEGs were identified in patients with SLE compared with HCs (**Figure 1C**), including 122 upregulated genes and 14 downregulated genes.

### Biological Functions of Common DEGs

GO and KEGG enrichment were performed on 136 overlapping DEGs (**Figures 1D, E** and **Supplementary Table 2**). According to GO enrichment, the biological process acted mainly on type I IFN signaling pathway and cellular response to type I IFN, and these proteins were mainly located in secretory granule membrane and lysosomal membrane. As to molecular functions, these proteins mainly took part in double-stranded RNA binding. Meanwhile, KEGG pathway analysis presented that these proteins were mainly involved in influenza A and proteasome signaling pathway.

### PPI Network Analysis, MCODE Cluster Modules, and Hub Gene Identification

**Figure 2:**
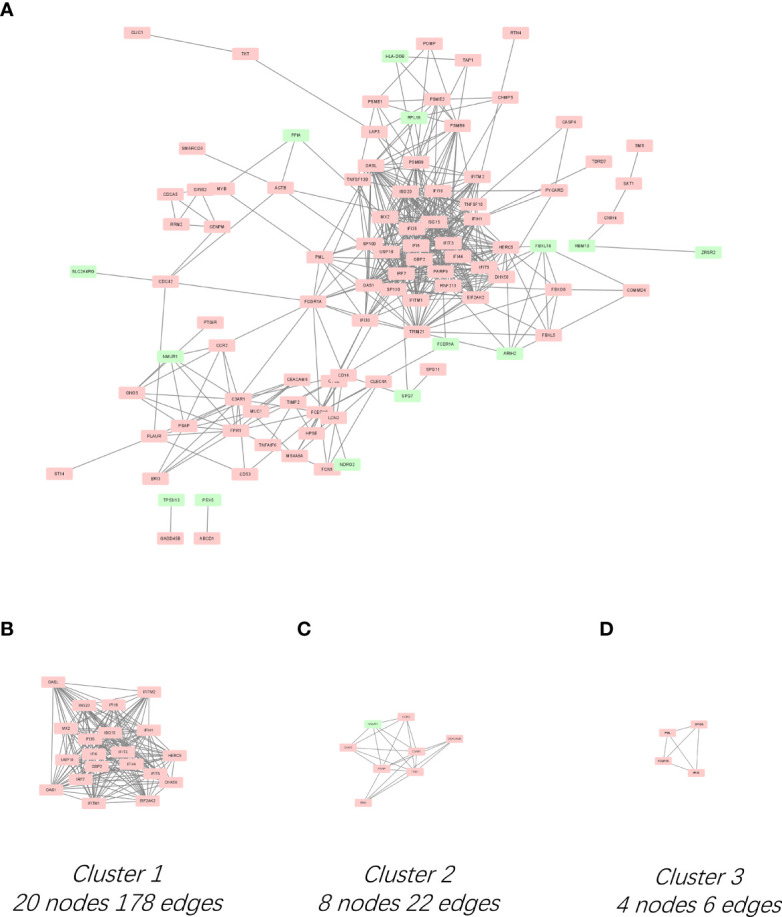
PPI network of DEGs and three cluster modules extracted by MCODE. **(A)** Interaction network between proteins coded by DEGs. Each node represents a protein, while each edge represents one protein–protein association. Red and green rectangles represent the upregulated and downregulated genes respectively. Three cluster modules extracted by MCODE. Cluster 1 **(B)** had the highest cluster score (MCODE score = 18.737), followed by cluster 2 **(C)** (MCODE score = 6.286), and cluster 3 **(D)** (MCODE score = 4).

The PPI network for the 136 DEGs was constructed after importing the common DEGs into STRING (**Figure 2A**). The top 10 hub genes were identified among DEGs based on the information in the STRING database and Cytoscape (**Supplementary Table 3**).The top 10 hub genes included IFN regulatory factor 7 (IRF7), IFN-induced 35 kDa protein (IFI35), IFN-induced protein with tetratricopeptide repeats 3 (IFIT3), ubiquitin-like protein ISG15 (IFN-induced 15 kDa protein, ISG15), 2′-5′-oligoadenylate synthase 1 (OAS1), IFN-regulated resistance GTP-binding protein MxB (MX2), 2′-5′-oligoadenylate synthase-like protein (OASL), IFN alpha-inducible protein 6 (IFI6), IFN-induced transmembrane protein 1 (IFITM1), and IFN-induced helicase C domain-containing protein 1(IFIH1) (**Supplementary Table 3**). All the top 10 hub genes were upregulated. The significant modules were identified by MCODE. MCODE score ≥4 was set as a threshold. Three modules with MCODE score≥4 are demonstrated in **Figures 2B–D**. Cluster-1 (MCODE score = 18.737) had 20 nodes and 178 edges (**Figure 2B**). Cluster-2 (MCODE score = 6.286) included eight nodes and 22 edges (**Figure 2C**). Cluster-3 (MCODE score = 4) consisted of four nodes and six edges (**Figure 2D**).

### The Expression Level of Ten Hub Genes Verified by Datasets GSE65391

The GSE65391 dataset from GPL10558 platform, which included 137 SLE samples and 53 HC samples with their follow-up data, was selected to verify the 10 identified hub genes. Ggplot2 and ggpubr packages were used to draw boxplots and perform the statistical analyses. The mRNA expression levels of the 10 hub genes in the SLE samples were significantly increased compared with those in the HC samples (P < 0.01, **Supplementary Figure 2A**).

### ROC Curves of 10 Hub Genes in SLE Samples

**Figure 3:**
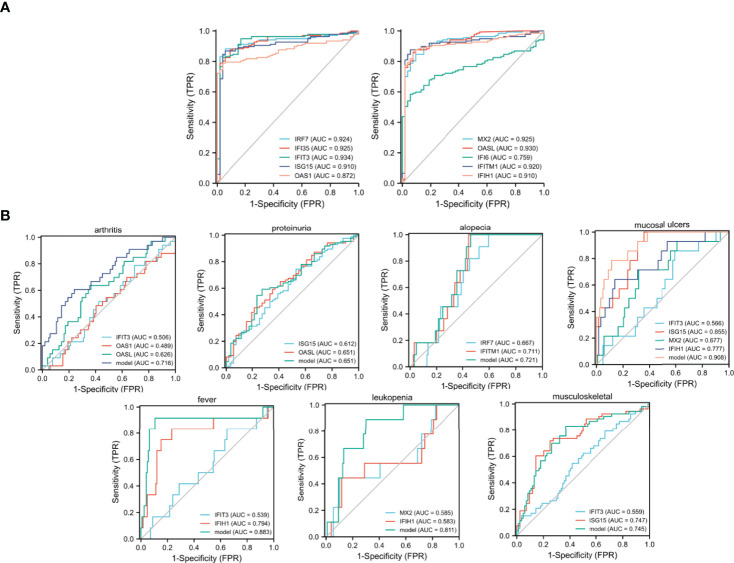
ROC curves generating by GSE65391. **(A)** ROC curves of the 10 identified hub genes in diagnosing SLE, **(B)** ROC curves of the identified hub genes in detecting different clinical manifestations of SLE.

The Series matrix file of GSE65391, offering the different expression levels of the identified hub genes, were imported into the RStudio. The software calculated the sensitivity, specificity, cut-off value, and AUC of the 10 hub genes (**Table 1**). The biomarker has good diagnostic accuracy with the AUC over 0.8, the sensitivity and specificity are over 80%. IFIT3 has the highest diagnostic value (AUC= 0.934). According to the results from our present samples, nine hub genes ((except IFI6)) may serve as promising biomarkers for the diagnosis of SLE (**Figure 3A**).

**Table 1:** The sensitivity and specificity of the 10 hub genes in detecting SLE.

Rank	Gene symbol	Sensitivity (%)	Specificity (%)	AUC(95% CI)	Cut-off value
1	IRF7	88.3	94.3	0.924 (0.876-0.971)	10.275
2	IFI35	87.6	90.6	0.925 (0.879-0.972)	8.985
3	IFIT3	82.5	96.2	0.934 (0.892-0.976)	8.657
4	ISG15	86.9	94.3	0.910 (0.861-0.959)	10.664
5	OAS1	78.8	96.2	0.872 (0.822-0.921)	7.891
6	MX2	84.7	90.6	0.925 (0.879-0.970)	9.968
7	OASL	86.1	92.5	0.930 (0.886-0.974)	5.596
8	IFI6	58.4	94.3	0.759 (0.693-0.825)	11.450
9	IFITM1	87.6	94.3	0.920 (0.873-0.967)	12.207
10	IFIH1	87.6	90.6	0.910 (0.860-0.959)	8.267

AUC, area under the curve; CI, confidence interval.

### Relationship Between Clinical Manifestations and Different Hub Genes

Multivariable logistic regression was conducted to verify the relationship between clinical manifestations and different hub genes (**Supplementary Table 4**). Seven clinical manifestations were significantly associated with the identified hub genes: arthritis and the levels of IFIT3 (odds ratio [OR] = 0.322, 95% confidence interval [CI] = 0.173-0.599; P < 0.01), OAS1(OR=0.699, 95%CI=0.503-0.971, P=0.033), and OASL (OR=3.4, 95%CI=1.454-7.948, P=0.005); proteinuria and the levels of ISG15(OR=0.49, 95%CI=0.212-1, P=0.05) and OASL (OR=2.029, 95%CI=1.013-4.016, P=0.046); alopecia and the levels of IRF7 (OR=0.047, 95%CI=0.003-0.885, P=0.041) and IFITM1(OR=31.168, 95%CI=1.342-723.877, P=0.032); mucosal ulcers and the levels of IFIT3 (OR=0.213, 95%CI=0.05-0.911, P=0.037), ISG15(OR=50.349, 95%CI=2.926-866.396, P=0.007), MX2 (OR=0.092,95%CI=0.01-0.813,P=0.032), and IFIH1; fever and the levels of IFIT3 and IFIH1(OR=9.726, 95%CI=1.613-58.658, P=0.013); leukopenia and the levels of MX2 (OR=0.013, 95%CI=0.001-0.235, P=0.003) and IFIH1 (OR=11.46,95%CI=1.039-126.436,P=0.046); and musculoskeletal disorder and the levels of IFIT3 (OR=0.462,95%CI=0.265-0.807, P=0.007) and ISG15 (OR=2.988, 95%CI=1.274-7.007, P=0.012).

### ROC Curves of the Identified Hub Genes in Detecting Different Clinical Manifestations of SLE

The ROC curves were shown to demonstrate the diagnostic efficacy of the identified hub genes in screening different clinical manifestations. The sensitivity, specificity, cut-off value, and AUC of the identified hub genes are listed in **Supplementary Table 5**. According to the results, the combination models of IFIT3, ISG15, MX2, and IFIH1 were effective in diagnosing mucosal ulcers among patients with SLE (AUC = 0.908, **Supplementary Table 5** and **Figure 3B**). The logistics regression model of the four hub genes was −37.226 + −1.0862 × IFIT3 + 3.6248 × ISG15 + −1.9284 × MX2 + 2.0384 × IFIH1. In addition, the combination model of IFIT3 and IFIH1 had a definite diagnostic accuracy in detecting fever among patients with SLE, and the regression model of the two hub genes was −18.6335 + −1.5369*IFIT3 + 3.2156 × IFIH1 (AUC = 0.883). The combination model of MX2 and IFIH1 was also good at determining the leukopenia group in patients with SLE, and the regression model was 5.6248 + −2.5919 × MX2 + 2.0122 × IFIH1 (AUC = 0.811).

### Correlation Between SLEDAI and Different Hub Genes

Linear correlation analysis was performed to clarify the relationship between SLEDAI and the expression of different hub genes. The results are shown in **Supplementary Figure 2B**. In the analysis process, seven hub genes, including IRF7, IFI35, ISG15, MX2, OASL, IFITM1, and IFIH1, were found to be statistically positively associated with SLEDAI (P < 0.005, **Supplementary Figure 2B**).

### Expression Levels of Hub Genes During Follow-Up

**Figure 4:**
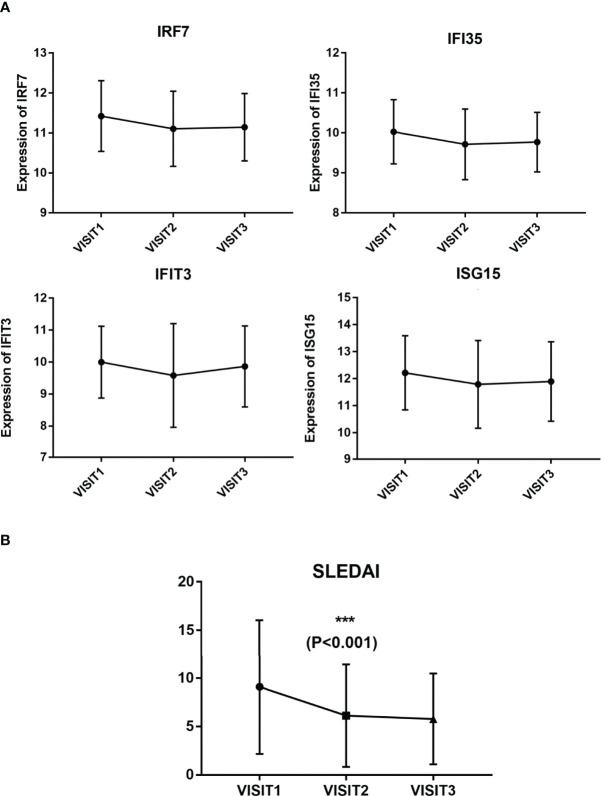
Expression levels of the four hub genes and SLEDAI during follow-up. **(A)** Expression levels of IRF7, IFI35, IFIT3, and ISG15 in three visits. **(B)** SLEDAI in three visits.

One-way repeated measures ANOVA was conducted to analyze the variation in the expression levels of hub genes during follow-up, and 69 patients were followed up three times. The gene expression levels of these 69 patients were analyzed. The expression levels of four genes, including IRF7, IFI35, IFIT3, and ISG15, were detected to be decreased at the second visit (not statistically, 0.005<P < 0.05, **Figure 4A**). SLEDAI of the patients during follow-up was remarkably decreased after treatment, as shown in **Figure 4B**. The mean expression levels of the 10 hub genes on each visit are listed in **Table 2**.

**Table 2:** The expression levels of hub genes during follow-up.

Hub gene	Visit 1 (mean ± SD)	Visit 2 (mean ± SD)	Visit 3 (mean ± SD)	P value
IRF7	11.42 ± 0.88	11.11 ± 0.94	11.15 ± 0.84	0.008
IFI35	10.03 ± 0.80	9.71 ± 0.88	9.77 ± 0.74	0.007
IFIT3	9.99 ± 1.12	9.58 ± 1.62	9,86 ± 1.26	0.032
ISG15	12.22 ± 1.37	11.79 ± 1.62	11.89 ± 1.47	0.024
OAS1	8.71 ± 1.58	8.72 ± 1.66	8.69 ± 1.58	0.962
MX2	10.69 ± 0.67	10.58 ± 0.72	10.59 ± 0.61	0.276
OASL	6.79 ± 1.21	6.59 ± 1.23	6.72 ± 1.91	0.298
IFI6	11.51 ± 1.00	11.39 ± 0.95	11.55 ± 0.99	0.434
IFITM1	12.89 ± 0.63	12.77 ± 0.59	12.67 ± 0.99	0.191
IFIH1	9.36 ± 1.04	9.15 ± 1.00	9.12 ± 0.87	0.195

## Discussion

SLE is one of the autoimmunity diseases with largely unknown molecular mechanisms (23). As the huge impact on patients’ quality of life, an urgent need for an enhanced understanding of the detailed mechanism to diagnose SLE and assess disease activity exists (24). In the present study, a series of bioinformatic analysis identified 10 hub genes (IRF7, IFI35, IFIT3, ISG15, OAS1, MX2, OASL, IFI6, IFITM1, and IFIH1) between SLE and HC samples based on the gene expression profiles obtained from the GSE49454 and GSE20864 datasets. The results were validated using the GSE65391 dataset. According to the results of ROC-AUC, the 10 hub genes had certain to good diagnostic performances. In addition, the combination model of different hub genes was good in defining different groups among patients with SLE. The expression levels of seven hub genes (except IFI6, OAS1 and IFIT3) were positively associated with disease activity. One-way repeated measures ANOVA revealed that the expression levels of four hub genes (IRF7, IFI35, IFIT3, and ISG15) decreased compared with the baseline expression levels (not remarkably, 0.005<P < 0.05). Furthermore, the biological functions of these common DEGs were investigated. Our GO analysis revealed that these DEGs were significantly associated with IFN response, in line with previous studies (25–27).

IRF7, a member of the IFN regulatory transcription factor family, is a key player in the regulation of many facets of innate immune responses (28). It is reported to be able to induce transcription of IFN-α and IFN-α-induced gene downstream of endosomal toll-like receptors (29). Available data have recently indicated that targeting TLR7-IRF7 signaling may be a promising treatment choice in SLE. One study reported a steady reduction in IRF7 mRNA gradually occurred after autologous hematopoietic stem cell transplant in patients with SLE, coinciding with the relatively low levels of IFN-regulated gene expression. Miyamoto et al. found that the nuclear translocation of IRF7 and IFN-α production in plasmacytoid dendritic cells could be suppressed by BAY11, one of the NF-kB inhibitors, in MRL-lpr mice (30, 31). Our study has detected that IRF7 had good diagnostic performance and was positively associated with disease activity. The expression level of IRF7 was negatively associated with the incidence of alopecia. Meanwhile, IRF7 was significantly decreased during patient follow-up. Therefore, IRF7 could function as a valuable marker for the diagnosis, assessment of disease activity, and treatment response of patients with SLE.

IFI35, IFIT3, IFITM1, and OASL, which are all proteins induced by IFN, have antimicrobial and innate defense abilities. IFI35 is associated with cellular proliferation, and IFIT3 is overexpressed in PBMCs of patients with SLE (32). Lihua Zhang et al. have reported that IFI35 enhanced the proliferation of mesangial cells in patients with lupus nephritis (33). A meta-analysis demonstrated that the gene expression of IFI35 was increased and hypomethylated in SLE and suggested to be a therapeutic target for SLE (34). In 2018, IFIT3 was found to be one of the genes that contribute to the overactive cGAS-STING signaling pathway in SLE monocytes, indicating that IFIT3 may serve as a therapeutic target to block the production of IFN and other proinflammatory cytokines generated by the cGAS-STING signaling pathway (35). The IFIH1 gene, better known as melanoma differentiation-associated gene 5, is located at 2q24.3, and it encodes a cytoplasmic dsRNA helicase belonging to the RLR family (36). A former study has demonstrated an association of IFIH1 rs1990760 polymorphism with SLE susceptibility in a Chinese population (37). OASL belongs to the 2-5-oligoadenylate synthetase (OAS) family (38). Similar to the present finding, a previous study reported upregulated OASL in patients with SLE. However, the exact relationship between OASL and disease activity has not been fully unveiled. ISG15, also related to enhanced type I IFN response, promotes the synthesis of IFNγ by T and B cells. One study recently showed that neutrophil extracellular traps from patients with SLE were characterized by the expression of ISG15 (39). Therefore, ISG15 in lupus pathogenesis may act as a durable proinflammatory stimulation. In the present study, IFI35, IFIT3, IFIH1, OASL, and ISG15, demonstrated good diagnostic ability, and they were associated with disease activity in patients with SLE. Moreover, IFI35, IFIT3, and ISG15 were discovered to be substantially decreased during patient follow-up. All these findings suggested that the IFN signaling pathway acted as a key factor in the pathological process of SLE.

Limited studies focused on the role of IFITM1 in SLE. Notably, IFITM1 plays an essential role in IFN-γ-induced cell proliferation of B-lymphocytes (40). IFITM1 is overexpressed in a large number of solid human tumors (40). The present study showed that IFITM1 has the ability to predict disease activity and identify patients with alopecia. Therefore, further studies are needed to discover its function in SLE pathogenesis.

Our study also demonstrated the relationship between clinical manifestations and different hub genes. Seven clinical manifestations were associated with different hub genes. Furthermore, the diagnostic efficacy of these identified hub genes in recognizing different clinical manifestations were tested with ROC-AUC. The combination model of IFIT3, ISG15, MX2, and IFIH1 was good in diagnosing mucosal ulcers among patients with SLE. The combination model of MX2 and IFIH1 could determine high-risk patients with leukopenia, and the combination model of IFIT3 and IFIH1 had a definite diagnostic accuracy in detecting fever among patients with SLE. These results might help us hierarchically manage patients.

The following limitations should be pay attention to in our study. On the one hand, we only focused on the hub genes, whereas the other DEGs, which may also have a diagnostic and therapeutic effect, were ignored. On the other hand, significance cutoff should be 0.005 after correction of multiple hypothesis testing for 10 genes. Therefore, none of the hub genes had significantly decreased during follow-up. Therefore, more precisely designed clinical studies are necessary to verify these potential genes.

In conclusion, on the basis of integrated bioinformatic analyses, differences in the biological functions in SLE compared with HC samples were identified. In particular, seven valuable genes, namely, IRF7, IFI35, ISG15, MX2, OASL, IFITM1, and IFIH1, were identified as potential biomarkers for the diagnosis and assessment of the disease activity of patients with SLE. Among them, IRF7, IFI35, IFIT3, and ISG15 decreased during the follow-up of patients with SLE (not significantly, 0.005<P < 0.05). In addition, the relationship between the expression levels of different hub genes and the clinical manifestations were investigated, and different combination models were effective in distinguishing patients with different features. Thus, the clinical values of the identified hub genes in SLE were demonstrated, and they might serve as a reference for future experimental research and clinical transformation.

In conclusion, on the basis of integrated bioinformatic analyses, differences in the biological functions in SLE compared with HC samples were identified. In particular, nine valuable genes, namely, IRF7, IFI35, IFIT3, ISG15, OAS1, MX2, OASL, IFITM1, and IFIH1, were identified as potential biomarkers for the diagnosis and assessment of the disease activity of patients with SLE. Among them, IRF7, IFI35, IFIT3, and ISG15 decreased during the follow-up of patients with SLE (not significantly, 0.005<P < 0.05). In addition, the relationship between the expression levels of different hub genes and the clinical manifestations were investigated, and different combination models were effective in distinguishing patients with different features. Thus, the clinical values of the identified hub genes in SLE were demonstrated, and they might serve as a reference for future experimental research and clinical transformation.

## Data Availability Statement

The datasets presented in this study can be found in online repositories. The names of the repository/repositories and accession number(s) can be found in the article/**Supplementary Material**.

## Author Contributions

LX designed the study. LX and FZ wrote the manuscript. SL revised the manuscript. All authors read and approved the final manuscript.

## Funding

This research was supported by Hainan Provincial Natural Science Foundation of China (820QN386). This project is supported by Hainan Province Clinical Medical Center.

## Conflict of Interest

The authors declare that the research was conducted in the absence of any commercial or financial relationships that could be construed as a potential conflict of interest.

## Publisher’s Note

All claims expressed in this article are solely those of the authors and do not necessarily represent those of their affiliated organizations, or those of the publisher, the editors and the reviewers. Any product that may be evaluated in this article, or claim that may be made by its manufacturer, is not guaranteed or endorsed by the publisher.
