# Supplementary material for: Clinical Values of the Identified Hub Genes in Systemic Lupus Erythematosus
Source: Front Immunol. 2022 Jun 9;13:844025. doi: 10.3389/fimmu.2022.844025 (PMC9219551; doi:10.3389/fimmu.2022.844025)
Supplement: Supplementary file 1 [file DataSheet_1.pdf]

Supplementary Material

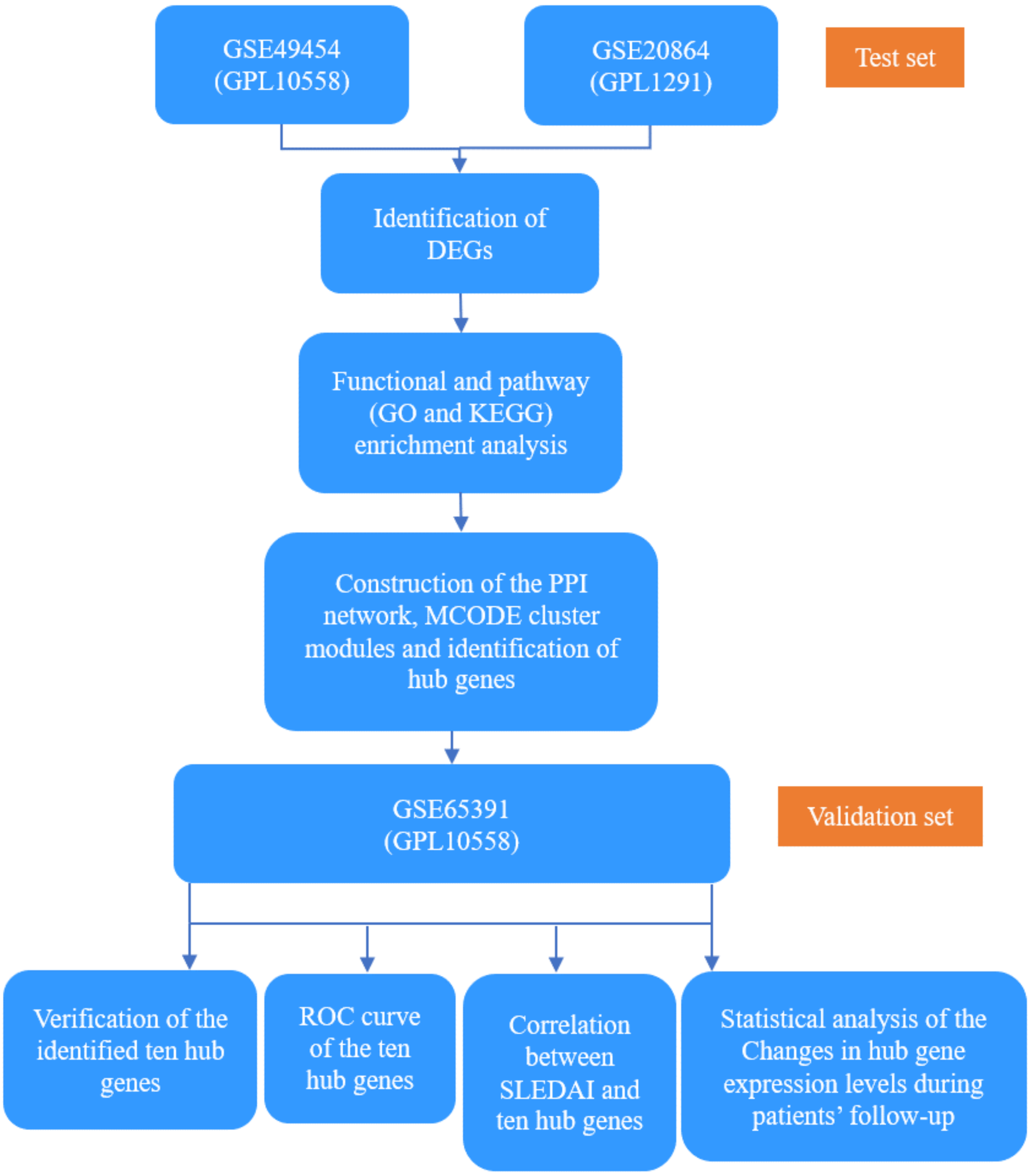

Supplementary Figure 1 Flowchart of the study.

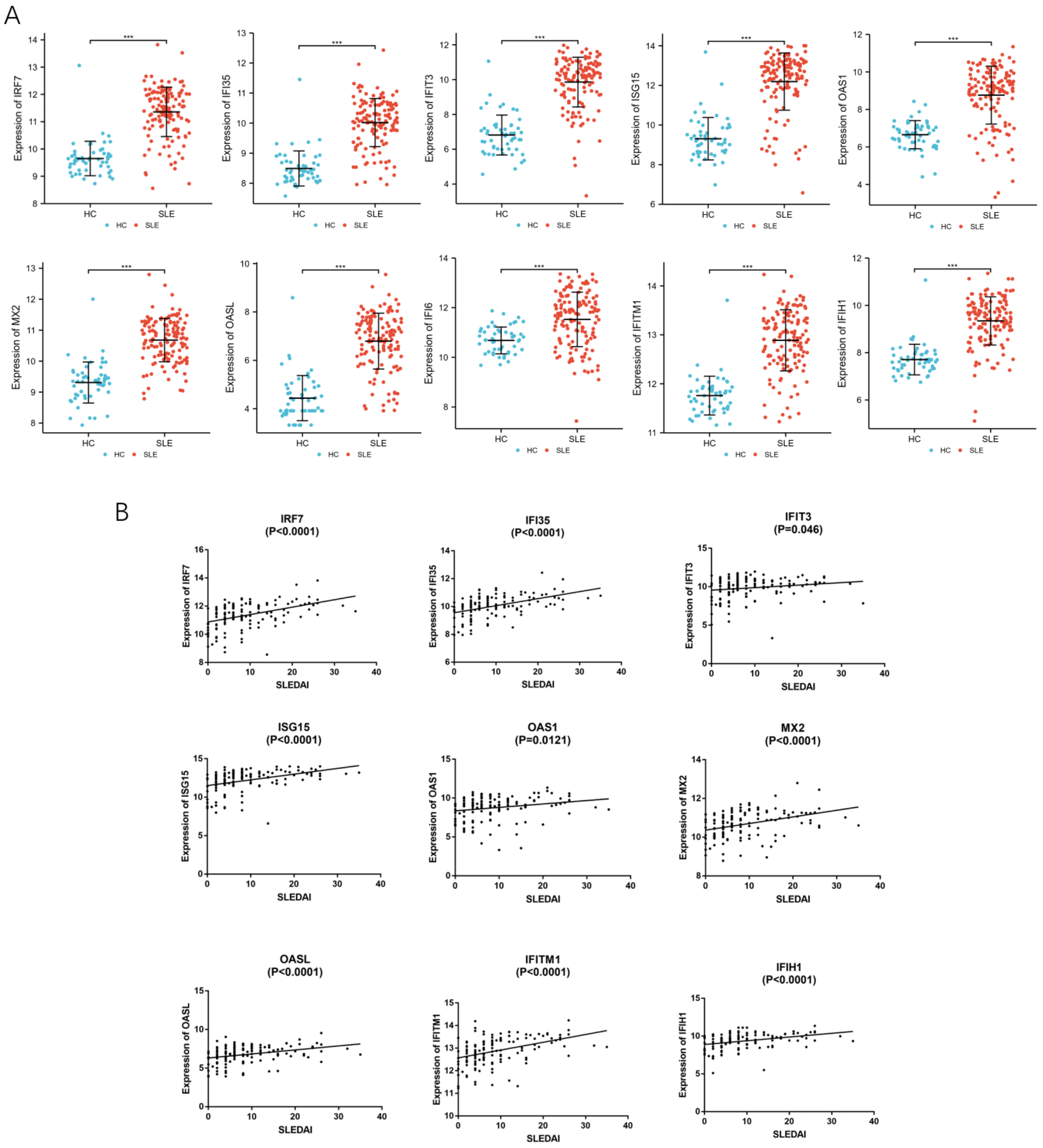

Supplementary Figure2 Verifications and linear correlation analysis of the identified hub genes by GSE65391 dataset.

(A) verification of the 10 identified hub genes. All hub genes are upregulated in SLE samples with significance. (B)Linear correlation analysis of nine hub genes. ***: P< 0.001.

Supplementary table

Supplementary Table 1 Information for selected microarray datasets

GEO accession	Platform	Samples		Source tissue	Age		Sex(male/female)		Attribute	Diagnostic criteria
		SLE	HC		SLE	HC	SLE	HC		
GSE49454	GPL10558	157	20	Whole blood	40.77	40.45	26/131	3/17	Test set	1997 ACR criteria for SLE(Hochberg, 1997)
GSE20864	GPL1291	21	45	Whole blood	38.95	35.35	0/21	23/22	Test set	1982 ACR criteria for SLE(Tan et al., 1982)
GSE65391	GPL10558	137	53	Whole blood	13.98	12.25	19/118	43/10	Validation set	Not mentioned

ACR: American College of Rheumatology

Supplementary Table 2 GO and KEGG analysis of DEGs

Ontology	ID	Description	GeneRatio	BgRatio	pvalue	p.adjust	qvalue
BP	GO:0060337	type I interferon signaling pathway	15/129	95/18670	1.17e-16	1.32e-13	1.15e-13
BP	GO:0071357	cellular response to type I interferon	15/129	95/18670	1.17e-16	1.32e-13	1.15e-13
CC	GO:0030667	secretory granule membrane	11/132	298/19717	5.35e-06	0.001	0.001
CC	GO:0005765	lysosomal membrane	11/132	354/19717	2.67e-05	0.002	0.002
MF	GO:0003725	double-stranded RNA binding	5/128	75/17697	2.11e-04	0.064	0.061
KEGG	hsa05164	Influenza A	10/72	171/8076	2.40e-06	3.75e-04	3.39e-04
KEGG	hsa03050	Proteasome	5/72	46/8076	5.05e-05	0.004	0.004

CC: cellular component group, MF: molecular function group, BP: biological process group, KEGG: Kyoto Encyclopedia of Genes and Genomes

Supplementary Table 3 10 hub genes identified by cytoHubba

Rank	Gene symbol	The name of protein	Regulation
1	IRF7	Interferon regulatory factor 7	UP
2	IFI35	Interferon-induced 35 kDa protein	UP
3	IFIT3	Interferon-induced protein with tetratricopeptide repeats 3	UP
4	ISG15	Ubiquitin-like protein ISG15 (Interferon-induced 15 kDa protein)	UP
5	OAS1	2'-5'-oligoadenylate synthase 1	UP
6	MX2	Interferon-regulated resistance GTP-binding protein MxB	UP
7	OASL	2'-5'-oligoadenylate synthase-like protein	UP
8	IFI6	Interferon alpha-inducible protein 6	UP
9	IFITM1	Interferon-induced transmembrane protein 1	UP
10	IFIH1	Interferon-induced helicase C domain-containing protein 1	UP

Supplementary Table 4 Multivariate logistic regression analyses of the relationship between clinical manifestations and different hub genes

	OR (95% CI)	P Value
Arthritis		
IFIT3	0.322(0.173-0.599)	<0.01
OAS1	0.699(0.503-0.971)	0.033
OASL	3.4(1.454-7.948)	0.005
Proteinuria		
ISG15	0.49(0.212-1)	0.05
OASL	2.029(1.013-4.061)	0.046
Alopecia		
IRF7	0.047(0.003-0.885)	0.041
IFITM1	31.168(1.342-723.877)	0.032
Mucosal ulcers		
IFIT3	0.213(0.05-0.911)	0.037
ISG15	50.349(2.926-866.396)	0.007
MX2	0.092(0.01-0.813)	0.032
IFIH1	9.726(1.613-58.658)	0.013
Fever		
IFIT3	0.189(0.065-0.549)	0.002

IFIH1	16.859(2.166-131.197)	0.007
Leukopenia		
MX2	0.013(0.001-0.235)	0.003
IFIH1	11.46(1.039-126.436)	0.046
Musculoskeletal		
IFIT3	0.462(0.265-0.807)	0.007
ISG15	2.988(1.274-7.007)	0.012

OR, odds ratio; CI, confidence interval.

Supplementary Table 5 The sensitivity and specificity of identified hub genes in detecting different clinical manifestations of SLE

	Sensitivity (%)	Specificity (%)	AUC (95% CI)	Cut-off value
Arthritis				
IFIT3	78.8	30.8	0.506 (0.394-0.617)	9.391
OAS1	48.5	59.6	0.489 (0.374-0.603)	9.253
OASL	63.6	63.5	0.626 (0.519-0.733)	7.163
Combination model	60.6	76	0.716 (0.612-0.819)	-0.982
Proteinuria				
ISG15	74.4	47.1	0.612 (0.514-0.711)	12.962
OASL	59.3	64.7	0.651 (0.556-0.746)	6.959
Combination	59.3	72.5	0.651 (0.556-0.746)	0.566
Alopecia				

IRF7	100	40.5	0.667 (0.554-0.781)	11.236
IFITM1	100	55.6	0.711 (0.602-0.820)	12.98
Combination model	100	54	0.721 (0.614-0.827)	-2.688
Mucosal ulcers				
IFIT3	85.7	40.7	0.566 (0.42-0.712)	9.933
ISG15	100	64.2	0.855 (0.773-0.937)	12.773
MX2	71.4	68.3	0.677 (0.533-0.822)	10.994
IFIH1	64.3	86.2	0.777 (0.637-0.917)	10.162
Combination model	78.6	88.6	0.908 (0.836-0.979)	-1.612
Fever				
IFIT3	83.3	35.2	0.539 (0.367-0.711)	10.564
IFIH1	83.3	76.8	0.794 (0.633-0.955)	9.911

Combination model	91.7	89.6	0.883 (0.736-1)	-1.478
Leukopenia				
MX2	44.4	89.8	0.585 (0.358-0.812)	9.91
IFIH1	44.4	88.3	0.583 (0.353-0.814)	10.358
Combination model	88.9	70.3	0.811 (0.689-0.933)	-2.615
Musculoskeletal				
IFIT3	54.7	58.3	0.559 (0.46-0.0.658)	10.284
ISG15	71.7	75	0.747 (0.659-0.834)	12.759
Combination model	83	63.1	0.745 (0.658-0.832)	-0.607

AUC, area under the curve; CI, confidence interval.

References:

Hochberg, M.C. (1997). Updating the American College of Rheumatology revised criteria for the classification of systemic lupus erythematosus. *Arthritis Rheum* 40(9), 1725. doi: 10.1002/art.1780400928.

Tan, E.M., Cohen, A.S., Fries, J.F., Masi, A.T., McShane, D.J., Rothfield, N.F., et al. (1982). The 1982 revised criteria for the classification of systemic lupus erythematosus. *Arthritis Rheum* 25(11), 1271-1277. doi: 10.1002/art.1780251101.
